# Prolonged time from intubation to cannulation in VV-ECMO for COVID-19: does it really matter?

**DOI:** 10.1186/s13054-021-03800-5

**Published:** 2021-11-10

**Authors:** Pierre-Yves Olivier, Gregoire Ottavy, Jerome Hoff, Johann Auchabie, Cedric Darreau, Marc Pierrot

**Affiliations:** 1 Medical ICU, Vent’Lab, University Hospital of Angers, Angers, France; 2 Centre Hospitalier Universitaire de Nantes, Département de Médecine Intensive Réanimation, Université de Nantes, Nantes, France; 3 Département de Médecine Intensive Réanimation, Centre Hospitalier de Saint Nazaire, Saint Nazaire, France; 4 Centre Hospitalier de Cholet, Réanimation et Unité de Surveillance Continue, Cholet, France; 5 Centre Hospitalier du Mans, Réanimation Médico Chirurgicale Et Unité de Surveillance Continue, Le Mans, France

Extrapolating data from H1N1 influenza pandemic, ELSO guidelines for VV-ECMO [1], consider a duration of mechanical ventilation exceeding 7 days as a relative major contraindication for VV-ECMO in patients with COVID-19-associated ADRS (CARDS).

The previously published cohorts of CADRS treated with VV-ECMO report a 65% survival rate with a strict patients’ selection [2]. We report the results of a retrospective cohort from three French ECMO centers, which did not apply such restrictive policy in these patients.

Data are presented as median value (interquartile range) or number (percentage). Comparisons were made using Wilcoxon–Mann–Whitney or Fisher’s exact tests as appropriate. Logistic models were used to evaluate associations after adjustment on covariates. According to French regulation, the study was approved by the Angers University Hospital ethics committee and information letters were sent to the patients.

Fifty-six patients (49 men, 7 women) aged 24 to 71 years were treated with ECMO from March 2020 to June 2021 (Table 1) after 0–36 days of mechanical ventilation. Initiation of ECMO was decided collegially, based on EOLIA trial criteria, namely refractory hypoxemia or hypercapnic acidosis (PaO_2_ < 80 mmHg for 6 h or < 60 mmHg for 3 h or pH < 7.25 for 3 h, despite appropriate ventilatory settings and prone positioning) [3], and depending on age, comorbidity and clinical history.

**Table 1:** Initial characteristics, respiratory care before VV-ECMO cannulation and evolution for COVID-19-associated ADRS

	Total cohort	ECMO survivors	ECMO non-survivors	*p*
	n = 56	n = 27	n = 29	
*General characteristics*				
Age (Years)	57 (51–65)	56 (50–60)	60 (55–66)	0.036
Male gender	49 (88)	23 (85)	26 (90)	0.7
BMI (kg/m^2^)	30 (27–34)	33 (29–36)	28 (27–34)	0.09
*Comorbidities*				
Hypertension	29 (52)	15 (56)	14 (48)	0.61
Diabetes	16 (29)	6 (22)	10 (35)	0.38
*Pre-ECMO care*				
Time from first symptom to ECMO (days)	18 (10–22)	18 (10–20)	18 (13–25)	0.22
Time from intubation to ECMO (days)	6 (4–13)	6 (1–13)	6 (4–10)	0.41
Prone position	56 (100)	–	–	–
Neuromuscular blockers	56 (100)	–	–	–
*Ventilation parameters at cannulation time*				
Tidal volume (mL/kg of PBW)	5.9 (5.5–6.1)	5.7 (5.4–6)	5.9 (5.5–6.1)	0.55
PEEP (cmH2O)	10 (6–14)	10 (7–14)	10 (5–14)	0.73
P Plat (cm H2O)	31 (30–34)	31 (30–35)	31 (30–32)	0.80
Compliance of RS (mL/cm H2O)	20 (15–23)	20 (14–24)	19 (15–24)	0.87
*Gazometric parameters at cannulation time*				
pH	7.29 (7.23–7.36)	7.30 (7.25–7.37)	7.28 (7.23–7.36)	> 0.99
PaO2/FiO2 (mm Hg)	62 (53–74)	60 (53–67)	62 (55–75)	0.38
PaCO2 (mm Hg)	63 (52–73)	64 (55–70)	62 (50–75)	> 0.99
*SOFA score at cannulation time*				
Total SOFA score	7 (4–8)	7(4–8)	7 (4–10)	0.75
SOFA score excluding respiratory item	3 (0–4)	3 (0–4)	3 (1–6)	0.49
*General evolution*				
ECMO duration (days)	18 (10–31)	18 (8–31)	17 (12–32)	0.85
ICU duration (days)	46 (28–60)	59 (47–75)	31 (23–43)	** < 0.01**
*ECMO complications*	29 (52)	–	–	–
Bleeding	48 (86)	20 (74)	28 (97)	**0.023**
Infections	37 (66)	18 (67)	19 (66)	> 0.99
Hemolysis	19 (34)	10 (37)	9 (31)	0.78
*Death causes n (% of non-survivors)*				
Bleeding	–	–	12 (41)	
Sepsis	–	–	10 (35)	
End-of-life decision	–	–	4 (14)	

Bold highlighted significant differences between the two groups (*p* < 0.05)

All data are presented as median (interquartile range) or number (percentage). Survivors and non-survivors are determined considering hospital mortality

The patients received VV-ECMO support for 17.5 (10–31.2) days and 27/56 (48%) discharged hospital. Patients’ survival according to age, SOFA score at cannulation and duration of mechanical ventilation before ECMO are reported in Fig. 1. Patients’ age was associated with mortality (*p* = 0.014). There was no significant association between the duration of mechanical ventilation before VV-ECMO and the mortality (mortality of 53% vs. 50% in patients cannulated before or after 7 days of mechanical ventilation, respectively, *p* > 0.999), even after adjustment on age and SOFA score at cannulation (adjusted OR = 0.76 [0.21–2.66], *p* = 0.673).

**Fig. 1:**
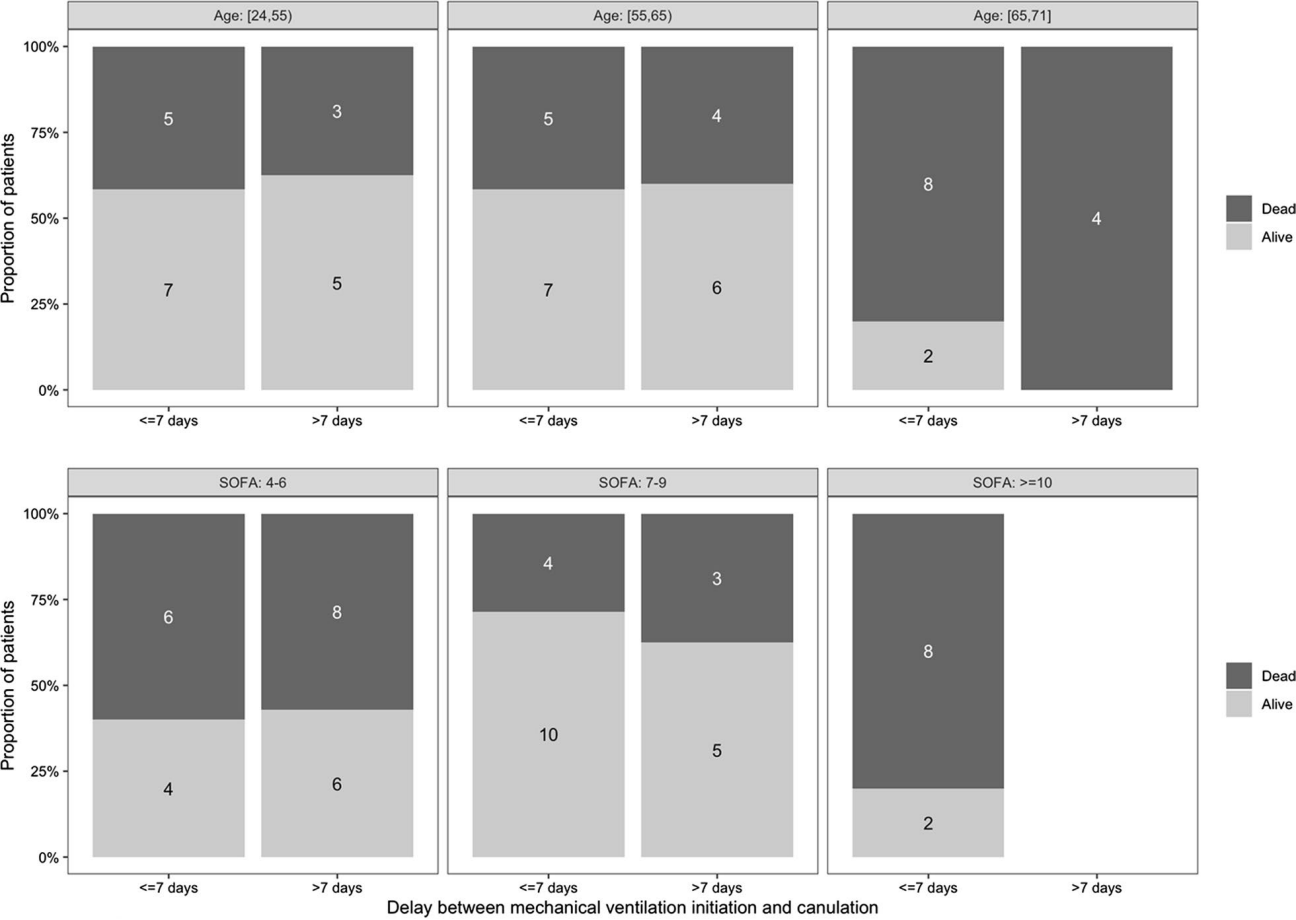
Hospital mortality according to age, SOFA score at cannulation time and duration of mechanical ventilation before VV-ECMO cannulation for COVID-19-associated ADRS. Twenty-seven out of 56 (48%) patients have survived with a strong correlation between age and mortality, especially if they were younger or older than 65 years (17/42 (40%) versus 12/14 (86%), respectively, *p* = 0.003). No correlation was found regarding the duration of mechanical ventilation before VV-ECMO cannulation and mortality or SOFA score at cannulation and mortality

The mortality observed in our cohort was higher than previously reported in patients treated with VV-ECMO during the first COVID-19 pandemic wave [2], including younger patients (median age of 49 vs. 57 years), but was similar to the one reported in larger cohorts of the second and third waves [4]. Our results are consistent with the data showing a strong association between age and mortality in COVID-19 patients [5].

The relative contraindication for VV-ECMO in patients mechanically ventilated for more than 7 days in ELSO guidelines remains questionable. It is mainly based on ELSO registry data referring to patients cannulated before 2012, particularly during the H1N1 influenza pandemic, reporting a strong correlation between mortality and duration of mechanical ventilation before cannulation [6], but has never been investigated in a prospective trial.

In the COVID-19 context with a very high pressure on available resources, most of the ECMO centers seem to have strongly limited their indications of late cannulation. To the best of our knowledge, our cohort is the first specific report of late cannulation experience in this indication and our results suggest that it is feasible and may have benefit some patients (69% survival rate in patients under 60 years of age cannulated after 7 days of mechanical ventilation in our cohort).

Our study has several limitations. It is a retrospective cohort with a limited number of patients, and some subjective criteria may have been considered in decisions of VV-ECMO initiation even if a predefined common protocol was used.

In conclusion, a late initiation of VV-ECMO for CARDS was not associated with an increased mortality in our cohort. The criterion of duration of mechanical ventilation for the decision of VV-ECMO initiation in this indication should be carefully considered.

## Data Availability

The datasets used and/or analyzed during the current study are available from the corresponding author on reasonable request.
